# Evidence for Introduction Bottleneck and Extensive Inter-Gene Pool (Mesoamerica x Andes) Hybridization in the European Common Bean (*Phaseolus vulgaris* L.) Germplasm

**DOI:** 10.1371/journal.pone.0075974

**Published:** 2013-10-01

**Authors:** Tania Gioia, Giuseppina Logozzo, Giovanna Attene, Elisa Bellucci, Stefano Benedettelli, Valeria Negri, Roberto Papa, Pierluigi Spagnoletti Zeuli

**Affiliations:** 1 Scuola di Scienze Agrarie, Forestali, Alimentari ed Ambientali, Università degli Studi della Basilicata, Potenza, Italy; 2 Dipartimento di Agraria, Sezione di Agronomia, Coltivazioni Erbacee e Genetica, Università degli Studi di Sassari, Sassari, Italy; 3 Dipartimento di Scienze Agrarie, Alimentari e Ambientali, Università Politecnica delle Marche, Ancona, Italy; 4 Dipartimento delle Scienze delle Produzioni Vegetali, del Suolo e dell'Ambiente Agroforestale, Università degli Studi di Firenze, Firenze, Italy; 5 Dipartimento di Biologia Applicata, Università degli Studi di Perugia, Perugia, Italy; 6 Consiglio per la Ricerca e la sperimentazione in Agricoltura, Cereal Research Centre (CRA-CER), Foggia, Italy; Nanjing Forestry University, China

## Abstract

Common bean diversity within and between Mesoamerican and Andean gene pools was compared in 89 landraces from America and 256 landraces from Europe, to elucidate the effects of bottleneck of introduction and selection for adaptation during the expansion of common bean (*Phaseolus vulgaris* L.) in Europe. Thirteen highly polymorphic nuclear microsatellite markers (nuSSRs) were used to complement chloroplast microsatellite (cpSSRs) and nuclear markers (phaseolin and *Pv-shatterproof1*) data from previous studies. To verify the extent of the introduction bottleneck, inter-gene pool hybrids were distinguished from “pure” accessions. Hybrids were identified on the basis of recombination of gene pool specific cpSSR, phaseolin and *Pv-shatterproof1* markers with a Bayesian assignments based on nuSSRs, and with STRUCTURE admixture analysis. More hybrids were detected than previously, and their frequency was almost four times larger in Europe (40.2%) than in America (12.3%). The genetic bottleneck following the introduction into Europe was not evidenced in the analysis including all the accessions, but it was significant when estimated only with “pure” accessions, and five times larger for Mesoamerican than for Andean germplasm. The extensive inter-gene pool hybridization generated a large amount of genotypic diversity that mitigated the effects of the bottleneck that occurred when common bean was introduced in Europe. The implication for evolution and the advantages for common bean breeding are discussed.

## Introduction

Common bean (*Phaseolus vulgaris* L., 2n = 2x = 22) is the most important edible food legume for direct human consumption in Europe and in the world as it represents a valuable source of proteins, vitamins, fibres, and minerals [1]. Genetic and archaeological studies have shown that domestication of *P. vulgaris* occurred as two distinct events in Mesoamerica and the Andes [2–5] resulting in two highly differentiated gene pools [3,6] that are characterized by geographical and partial reproductive barriers [7,8]. Evidence supporting the divergence of these two major domesticated gene pools was based on morphological characters [3,9,10], agronomic traits [10], seed proteins [11], allozymes [2], and a number of molecular markers, including RFLP [4], RAPD [12], AFLP [13,14], SSRs [15], cpSSRs [16], and DNA sequences [17]. The divergence between the Andean and Mesoamerican gene pools has important implications for bean breeding. Despite their partial reproductive isolation [7,8], the two gene pools still belong to the same biological species. Viable and fertile progeny can be obtained by inter-gene pool crosses, and therefore, genes can be transferred between the two pools, although the transfer of quantitative traits appears to be problematic [18].

Common bean was brought to Europe after the first voyages of Columbus (1492) but historical and linguistic sources provide little evidence of the introduction and expansion of common bean in Europe. When phaseolin type is used to trace the origin of accessions to the Middle American or Andean domestication centers, their distribution patterns show that the two American gene pools were introduced into Europe at different times [18–21]: the Mesoamerican probably through Spain and Portugal in 1506 [22], and the Andean after the exploration of Peru by Pizarro in 1528 [23]. Both common bean gene pools spread widely in all parts of Europe with very complex pathways of dissemination that included several introductions from various regions of the Americas, combined with direct exchanges between European and other Mediterranean countries (see Papa et al. [24], for a review). In Europe the frequency of Andean phaseolin types (76%) was higher than Mesoamerican types (24%) [25], as it was confirmed by Lioi [26] (66%) in an analysis of a large collection from Italy, Greece and Cyprus, by Logozzo et al. [27] (76%) in a European core collection, by Rodinõ et al. [28] and Ocampo et al. [29] for Portuguese and Spanish samples, and by others at a regional scale [21,30–33]. During the five centuries since common beans were introduced into Europe, many landraces and varieties evolved under diverse environments and farmer preferences, to provide dry seed or fresh pods [20]. It was expected that introduction from America into Europe might have caused a loss of variation due to the introduction bottleneck and to selection for adaptation to new environments and consumer preferences [34]; but, recent studies using phaseolins, allozymes and morphological data [18,35], ISSRs and SSRs from both the chloroplast and nuclear genomes [16,32,36], have suggested that the reduction of variation might have been less than previously suspected and that hybridization that occurred in Europe between the Andean and Mesoamerican gene pools probably had a significant impact on the maintenance of the overall level of genotypic diversity.

Logozzo et al. [27] first noted that European accessions with phaseolin “S” (Mesoamerican) showed a significantly larger seed size than those of the same phaseolin class in America while those with phaseolin “T” and “C” did not, suggesting a different contribution of the two gene pools to the genetic structure of the European germplasm, and the possible contribute of inter-gene pool hybridization. Recently, Angioi et al. [16], combining the information provided by six chloroplast (cpSSR) and two nuclear markers (phaseolin and *Pv-shatterproof1*) observed that 33.1% of the European bean germplasm was derived from Andean x Mesoamerican hybridization. Using a maximum likelihood approach, they also estimated that 11.2% of the European individuals might be “hidden” hybrids that are observed as “pure”, and thus they predicted that about 44% of the European bean germplasm is derived from inter-gene pool hybridization. Nevertheless, their hypothesis should be supported by using more nuclear data that discriminate between the two gene pools.

In common bean a large number of nuclear microsatellite markers (nuSSRs) have been already developed and mapped [37–42] that show relatively high levels of polymorphism, thus providing an attractive choice for describing population structure. However, to the best of our knowledge, population studies of the European common bean, using nuSSRs, so far have been performed with only a small number of landraces or a small number of samples from a few geographic regions [32,43–45].

The aim of the work was: (i) to study the patterns of diversity for nuSSR markers within a European “core collection” of *P. vulgaris* L. [27] that included both the Mesoamerican and Andean gene pools, and to compare the results with a representative sample of American landraces of the same gene pools; (ii) to identify the inter-gene pool hybrids combining Bayesian analysis of nuSSRs with gene pool recombination for three markers (cpSSR, phaseolin and *Pv-shatterproof1*) that are specific for each the two genepools; (iii) to estimate the effects of genetic bottleneck following the introduction into Europe, by distinguishing “pure” and hybrid accessions.

## Materials and Methods

### Plant material

A total of 345 common bean accessions were studied: 256 landraces from Europe and 89 from the Americas. These genotypes represent most of the germplasm formerly investigated with phaseolin and morphological seed traits [27] and with cpSSRs [16]. The European material represents a large proportion of 300 accessions of a common bean “core collection” [27] that includes all European countries, and was developed using a sampling strategy stratified by the logarithm of frequency of phaseolin types. The “core collection” was validated using seven morphological seed characters. The European accessions were compared with 89 American domesticated common bean entries from Central America and South America that are representative of the Andean and Mesoamerican gene pools (Table S1).

In America, the geographic origin of individual accessions is not a reliable indicator of the origin of domestication, because of the exchange between the Andean and Mesoamerican gene pools after domestication, and the subsequent dissemination across different regions [6,11]. Gene pool assignment in European germplasm was mostly based on phaseolin seed protein types, with “T” and “C” types belonging to the Andean gene pool and the “S” type belonging to the Mesoamerican gene pool [18,28]. Since recombination events appeared to be frequent, the assignment based on phaseolin type was likely unreliable thus, the gene pool designation was based on the highly reliable STRUCTURE analysis for K = 2 [46]. According to this information, the 256 European accessions were identified as 173 Andean and 83 Mesoamerican, while the 89 American accessions were 43 Andean and 46 Mesoamerican (Table S1).

All the accessions were obtained from the Institute of Plant Genetics and Crop Plant Research (IPK), Germany; Centro Internacional de Agricoltura Tropical (CIAT), Colombia; Centro per la Salvaguardia delle Risorse Genetiche Vegetali “Pierino Iannelli”, Università della Basilicata (CUB), Italy; the United States Department of Agriculture (USDA), USA; the Department of Scienze Ambientali e delle Produzioni Vegetali (SAPROV) of the Università Politecnica delle Marche (Univ. P.M.), Italy; the Nordic Gene Bank (NGB), Sweden; Plant Genetic Resources in Czech Republic (Evigez), Czech Republic; and the Department of Plant Science, UC, Davis, USA. A complete list of the accessions studied, along with information on their origins and with assigned gene pool and posterior membership coefficients as determined with STRUCTURE is available in Table S1.

### Genomic DNA extraction and genotyping microsatellites

Genomic DNA was extracted from young trifoliate leaves of one individual 10-day-old greenhouse-grown plant per accession using the CTAB method [47] with minor modifications. Twenty nuSSRs from all 11 common bean linkage groups were chosen based on their dispersed map locations [37,40]. All of these were developed from genomic sequences, and were located on the consensus genetic map of *P. vulgaris* constructed using a RIL population obtained from the cross between the Mesoamerican cultivar BAT93 and the Andean cultivar Jalo EPP558 [37,40]. The primers were initially tested on a sub-sample of 20 accessions. Of the initially tested markers, 13 polymorphic nuSSRs that generated distinct amplification products were used to assay genetic diversity within the whole collection. More information about the nuSSR markers used, including the primer pair sequences and repeat motif can be found in Table 1.

**Table 1:** Primers sequence and characteristics of the 13 nuSSR markers used to study diversity in a germplasm collection of Andean and Mesoamerican common bean from Europe and America.

**Linkage Group**	**Marker**	**GenBank entry**	**Reference**	**T_a_ (°C)**	**Expected product size**	**Primer sequence (5'–3')**	**Repeat motif**	**Number of alleles**	**Gene diversity**	**PIC**
1	BMd45	AF293023	Blair et al. 2003	47	129	F: GGTTGGGAAGCCTCATACAG	(AG)_5_	3	0.491	0.373
						R: ATCTTCGACCCACCTTGCT				
2	PVcct001	X79722	Yu et al. 2000	52	149	F: CCAACCACATTCTTCCCTACGTC	(CCT)_7_	3	0.275	0.243
						R: GCGAGGCAGTTATCTTTAGGAGTG				
3	BMd1	X96999	Blair et al. 2003	52	165	F: CAAATCGCAACACCTCACAA	(AT)_9_	10	0.805	0.779
						R: GTCGGAGCCATCATCTGTTT				
4	PVat003	X60000	Yu et al. 2000	50	163	F: ACCTAGAGCCTAATCCTTCTGCGT	(AT)_4_ (T)_2_ (AT)_6_	3	0.536	0.430
						R: GAATGTGAATATCAGAAAGCAAATGG				
4	PVag004	X04660	Yu et al. 2000	52	201	F: TTGATGACGTGGATGCATTGC	(AG)_8_	13	0.780	0.755
						R: AAAGGGCTAGGGAGAGTAAGTTGG				
5	PVat006	X74919	Yu et al. 2000	52	132	F: CCGTTGCCTGTATTTCCCCAT	(AT)_5_	5	0.602	0.552
						R: CGTGTGAAGTCATCTGGAGTGGTC				
6	BMd12	AZ044945	Blair et al. 2003	47	167	F: CATCAACAAGGACAGCCTCA	(AGC)_7_	2	0.414	0.328
						R: GCAGCTGGCGGGTAAAACAG				
7	PVatcc001	J01263	Yu et al. 2000	52	171	F: ATGCATGTTCCAACCACCTTCTC	(ATCC)_3_ (AG)_2_ (TAC)_3_	3	0.051	0.050
						R: GGAGTGGAACCCTTGCTCTCATC				
8	BMd44	AZ301573	Blair et al. 2003	47	135	F: GGCAGCTTACTAACCCGAAA	(AG)_5_	2	0.499	0.374
						R: TTCCTTCCCCTTTCTTCTCC				
10	BMd41	AZ301561	Blair et al. 2003	47	250	F: CAGTAAATATTGGCGTGGATGA	(ATT)_9_	3	0.575	0.509
						R: TGAAAGTGCAGAGTGGTGGA				
10	BMd42	AZ301511	Blair et al. 2003	47	149	F: TCATAGAAGATTTGTGGAAGCA	(AT)_5_	5	0.703	0.656
						R: TGAGACACGTACGAGGCTGTAT				
11	PVag001	m75856	Yu et al. 2000	50	157	F: CAATCCTCTCTCTCTCATTTCCAATC	(CT)_11_	3	0.641	0.565
						R: GACCTTGAAGTCGGTGTCGTTT				
11	BMd43	AZ301513	Blair et al. 2003	47	176	F: CAGCATCAAGAAGACCCAAG	(CCT)_5_	4	0.501	0.381
						R: CAGCACCACTATGGGAGGAC				
	Mean							4.54	0.529	0.461

Polymerase chain reaction (PCR) amplifications were performed in a 25 µl reaction volume, containing 25 ng template DNA, 10 pmol of each primer, 20 µM dNTPs, PCR buffer 1X (200 mM Tris–HCl, pH 8.4, 500 mM KCl), 50 mM MgCl_2_ and 1U Taq polymerase (Invitrogen). The amplifications were carried out with a PerkinElmer GeneAmp 9700 thermocycler (Applied Biosystems), using different annealing temperature conditions depending on primer pair. For PVatcc001, PVcct001, PVag001, PVat003, PVag004, BMd1, and PVat006, amplifications were conducted under the following conditions: 15 min at 95°C; 35 cycles of 10 sec at 92°C, 10 sec at 50-52°C, 2 min at 72°C; 30 sec at 72°C. For the other 6 nuSSR loci (BMd44, BMd43, BMd42, BMd41, BMd12, and BMd45), the PCR cycle consisted of 15 min at 95°C; 35 cycles of 1 min at 92°C, 1 min at 47°C, 2 min at 72°C; 5 min at 72°C. DNA fragments were separated in 6% 8 M denaturing acrylamide:bisacrylamide (19:1) gels run at 70 W for 3 h in a vertical cell (Biorad Laboratories, Milan, Italy), and visualized using the silver staining method [48].

To obtain a better picture of the genetic variation observed in European *P. vulgaris*, and to verify the ability of different markers to identify putative inter-gene pool hybrids that probably have different degrees of introgression, six cpSSR markers and two unlinked nuclear loci (for phaseolin types and *Pv-shatterproof1*), available from Angioi et al. [16], were also used. By combining nuSSRs with the haploid cpSSRs, demographic processes acting on different time scales will be captured, because of the different modes of inheritance of nuclear markers, effective population size and mutation rate [49]. Information on typing chloroplast haplotypes, phaseolin type and *Pv-shatterproof1* locus are accessible from Angioi et al. [16].

### Statistical analysis

For each nuSSR locus, the total number of alleles detected, the gene diversity or unbiased expected heterozygosity (H_e_ [50]), and the polymorphism information content (PIC) were calculated using the program Power Marker 3.25 [51].

The genetic diversity within continents (America and Europe), within the two gene pools (Andean and Mesoamerican), and within gene pools within continents (America Andean, America Mesoamerican, Europe Andean, and Europe Mesoamerican) was evaluated in terms of number of alleles per locus (N_a_), Shannon diversity index (I), and gene diversity or unbiased expected heterozygosity (H_e_ [50]). Number of private alleles was also computed using a threshold frequency of 5% to reduce the effects of sampling error [52]. All these indices were calculated using GenAlEx 6 [53]. As the number of alleles in a sample is highly dependent on the sample size, we also computed the allelic richness (R_s_) using the generalized rarefaction method as implemented in HP-RARE [54]. HP-RARE uses the rarefaction approach of Kalinowski [55] to trim unequal accession number to the same standardized sample size, a number equal to the smallest across the populations. Relative loss of diversity in terms of alleles (ΔR_s_) and genetic diversity (ΔH_e_) was calculated according to Vigouroux et al. [56]. Differences between populations on the gene diversity estimates were assessed for significance using Wilcoxon’s signed-rank test as implemented in the software StatistiXL (http://www.statistixl.com).

To further investigate the genetic relationships between all pairs of accessions, an individual-by-individual (*N x N*) genetic distance matrix was computed and subsequently used as an input for principal coordinate analysis (PCoA) in the GenAlEx 6 program [53].

Pairwise F_ST_ metrics were calculated in GenAlEx 6 to estimate the divergence between groups according to the formula of Weir and Cockerham [57]. The value of F_ST_ varies from zero to one; when F_ST_ = 0, the groups are identical, while when F_ST_ = 1, they are completely differentiated in relation to the fixation of different alleles in each group.

To assess the distribution of genetic variations in the nuSSR dataset, a hierarchical analysis of molecular variance (AMOVA) was also performed, using GenAlEx 6 [53]. This analysis allowed the partition of the total nuSSR variation into within and among groups variance components, and provided measures of intergroup genetic distance as a proportion of the total nuSSR variation residing between any two groups (Phi statistics [58]). Genetic variation was partitioned into three levels: between continents (America and Europe), between gene pools (Mesoamerican and Andean) within continent and within gene pool within continent. The significance of the variance components and the differentiation statistic were tested by nonparametric randomization tests using 10,000 permutations.

A Bayesian clustering approach, implemented in STRUCTURE 2.2 [46], was adopted to first assess the number of meaningful populations (K) and second to identify putative inter-gene pool hybrids within our collections, with no “a priori” information other than nuSSR genotype data. The STRUCTURE program was run with populations (K) set from one to ten. Twenty independent simulations were performed for each K setting using the admixture model, with each simulation set to a 5,000 burn-in period and 50,000 Markov chain Monte Carlo (MCMC) repetitions. To determine the optimal number of clusters, STRUCTURE HARVESTER [59], available at http: //taylor0.biology. ucla.edu/struct_harvest/, was used to calculate the ΔK statistical test [60], in combination with the likelihoods (posterior probabilities) of each preset K. Results from simulations with the highest likelihood within each number of different K simulations were chosen to assign accessions to populations. Following the recommendation of Pritchard et al. [46], and previous useful analysis in common bean [15,44], accessions with population membership coefficient lower than 0.8 were identified as putative hybrids. A STRUCTURE graphical bar plot of membership coefficients was generated using Microsoft Excel.

Hybridization between the Mesoamerican and Andean gene pools in Europe and America was also investigated by combining the information provided by chloroplast (cpSSR) and nuclear (phaseolin, *Pv-shatterproof1*) markers with the Bayesian assignments based on nuSSRs. Genotypes were classified as hybrids if the chloroplast or any of the two nuclear markers did not agree with the STRUCTURE gene pool assignment (i.e. a genotype was attributed to the Andean gene pool but had Mesoamerican “S” phaseolin type). To validate the levels of genetic admixture in the common bean, we then compared our results with hybrid identification according to [16] as recombinant for chloroplast (cpSSR) and nuclear (phaseolin, *Pv-shatterproof1*) markers. In this approach, if the genetic patterns of variation in chloroplast and nuclear markers resulting from the analysis of recombinant were concordant (i.e. an individual is attributed to the Andean gene pool, or Mesoamerican gene pool by all of these marker types), then the accessions were classified as “pure”. On the contrary, accessions with a mismatch between their chloroplast and nuclear polymorphisms were classified as putative hybrids.

## Results

### Overall nuSSRs diversity

The amount of polymorphism in terms of mean number of observed alleles, expected heterozygosity, and PIC values for each of the 13 nuSSRs evaluated is reported in Table 1. A total of 59 alleles were observed across the full set of genotypes. The average number of alleles per microsatellite was 4.54, and ranged from 2 alleles (BMd12 and BMd44), to 13 alleles (PVag004). Gene diversity, or expected heterozygosity (H_e_), across all the accessions was 0.529. The PIC values, a reflection of allele diversity and frequency, were 0.461 for all the microsatellites, and ranged from a low 0.050 (PVatcc001) to a high 0.779 (BMd1) (Table 1).

### nuSSRs genetic diversity

Our primary objective was to provide an overview of genetic variation detected by nuSSR markers within and amongst the germplasm from Europe and the Americas for both the Andean and the Mesoamerican gene pools. STRUCTURE data analysis for K = 2 was used to identify the two major gene pools in the study sample (Table S1).

The intra-population genetic diversity measures are shown in Table 2a. A significantly higher (Wilcoxon signed-rank test, *P* < 0.001) number of total alleles was observed in America (N_a_ = 55), compared to Europe (N_a_ = 48). Similarly, the allelic richness and the Shannon diversity index in America were significantly higher than those observed in Europe (Wilcoxon signed-rank test, *P* < 0.001 for both allelic richness and Shannon diversity index, respectively). The relative deficit in allele number ΔR_s_ was 0.16, meaning that European common bean has 16% fewer alleles than American common bean. In contrast with allelic richness, the gene diversity was not significantly different (Wilcoxon signed-rank test, *P* > 0.05) in America as compared to Europe (H_e_ = 0.52 vs. H_e_ = 0.51) (Table 2a). This leads to positive but small values of ΔH_e_ (0.02). We also computed the number of private alleles, imposing the allele frequency threshold of 5%, in order to reduce chances of confounding allele classification with sampling error [52]. No private alleles were found in Europe, while America showed four private alleles.

**Table 2:** Summary statistics of diversity for 13 nuSSR markers in a) 345 Andean and Mesoamerican accessions of common bean from America and Europe and b) in ‘pure’ (not admixed) Andean and Mesoamerican common bean accessions as identified on the basis of both chloroplast and nuclear markers and by STRUCTURE analysis.

**Gene pool**	a) **All European & American accessions**							b) **Only American & European “pure” accessions**						
	**n**	**N_a_**	**R_s_**	**ΔR_s_**	**I**	**H_e_**	**ΔH_e_**	**n**	**N_a_**	**R_s_**	**ΔR_s_**	**I**	**H_e_**	**ΔH_e_**
*American*														
Andean	43	43	3.16		0.65	0.33		37	41	3.01		0.65	0.33	
Mesoamerican	46	36	2.61		0.56	0.31		41	36	2.61		0.56	0.31	
America all	89	55	4.07		0.97	0.52		78	52	3.94		0.94	0.52	
*European*														
Andean	173	44	3.03	*0.04*	0.67	0.36	*-0.08*	102	41	2.87	*0.05*	0.61	0.31	*0.06*
Mesoamerican	83	42	2.99	*-0.13*	0.56	0.29	*0.06*	51	33	2.36	*0.10*	0.41	0.22	*0.29*
Europe all	256	48	3.41	*0.16*	0.89	0.51	*0.02*	153	47	3.43	*0.13*	0.90	0.51	*0.02*
Andean all	216	51	3.63		0.69	0.36		139	44	3.29		0.64	0.33	
Mesoamerican all	129	47	3.52		0.63	0.34		92	41	3.03		0.57	0.31	
*Total*	345	59	–		0.93	0.52		231	49	–		0.92	0.52	

n, number of samples; Na, total number of alleles; R_s_, allelic richness; ΔR_s_, loss of alleles calculated as ΔR_s_ = 1- (R_s_ Europe/ R_s_ America), if R_s_ Europe > R_s_ America the parameter was calculated as ΔR_s_ = - [1- (R_s_ America/ R_s_ Europe)]; I, Shannon’s information index; H_e_, expected heterozygosity; ΔH_e_, loss of genetic diversity calculated as ΔH_e_ = 1- (H_e_ Europe/America), if H_e_ Europe > H_e_ America the parameter was calculated as ΔH_e_ = - [1- (H_e_ America/ H_e_ Europe)].

Estimates of the number of total alleles, allelic richness and gene diversities for the American and European common bean within the Andean and the Mesoamerican gene pools are also presented in Table 2a. In the Andean gene pool, America displayed a significantly higher allelic richness per locus (Rs = 3.16) than Europe (Rs = 3.03) (ΔR_s_ = 0.04). In contrast, the number of alleles, the Shannon diversity index and gene diversity were not significantly different (Wilcoxon signed-rank test, *P* > 0.05) in America (N_a_ = 43, I = 0.65, H_e_ = 0.33) as compared to Europe (N_a_ = 44, I = 0.67, H_e_ = 0.36) (ΔH_e_ = -0.08) (Table 2a). Only 1 and 4 private alleles were found in the American and European Andean populations, respectively.

In the Mesoamerican gene pool, the number of alleles and the allelic richness per locus were slightly higher in Europe than in America (Table 2a). Therefore, the relative deficit in allele number ΔR_s_ was negative (ΔR_s_ = -0.13). In contrast, with allelic richness, the gene diversities was slightly higher but not significant (Wilcoxon signed-rank test, *P* > 0.05) in America than in Europe (H_e_ = 0.31 vs. H_e_ = 0.29). This leads to positive but small value of ΔH_e_ (0.04) (Table 2a).

### nuSSRs genetic relationships within and between Europe and America

**Figure 1:**
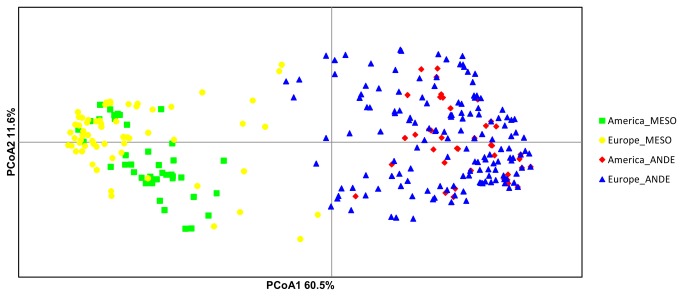
Principal coordinate analysis (PCoA) of nuSSRs diversity among 345 Andean and Mesoamerican accessions of common bean from America and Europe. The samples are color coded according to their gene pool and the continent of origin as identified by Bayesian STRUCTURE analysis.

A visual representation of genetic relationships between common bean populations based on the principal coordinate analysis (PCoA) is reported in Figure 1. The first three principal coordinates accounted for 81.1% of the total variance detected, comprising 60.5% the first (PC1), 11.6% the second (PC2) and 9.0% the third (PC3) principal coordinate. In the scatter diagram using the first two components, the samples are color-coded according to STRUCTURE assignment. The Andean and the Mesoamerican gene pools are clearly distinct along the first axis (PC1). Furthermore, the separation between American and European accessions was stronger within the Mesoamerican than within the Andean gene pool cluster. Based on the placement of accessions along the first PCoA, a number of European accessions map in between the two major Andean and Mesoamerican groups, possibly showing inter-gene pool genetic admixture.

### nuSSRs genetic differentiation between Europe and America

Overall, the differentiation between continents (America vs. Europe) based on F_ST_ was very low, but significant (F_ST_ = 0.049; *P* < 0.001) (Table 3a). As expected, a high significant genetic differentiation was detected between the Andean and the Mesoamerican gene pools (F_ST_ = 0.520; *P* < 0.001). The two gene pools also showed high divergence within each continent, with highly significant (*P* < 0.001) F_ST_ values in America (F_ST_ = 0.547) and Europe (F_ST_ = 0.540). The degree of differentiation between America and Europe was moderate for the Mesoamerican gene pool (F_ST_ = 0.186) and very small for the Andean gene pool (F_ST_ = 0.026).

**Table 3:** F_ST_-based genetic differentiation for 13 nuSSR markers in a) 345 Andean and Mesoamerican accessions of common bean from America and Europe and b) in ‘pure’ (not admixed) Andean and Mesoamerican common bean accessions as identified on the basis of both chloroplast and nuclear markers and by STRUCTURE analysis.

**Gene pool**	**Differentiation level**	**SSR mean F_ST_**	
		**a) All accessions**	**b) Only “Pure” accessions**
Overall	American vs. European	0.049***	0.052***
Overall gene pools	Andean vs. Mesoamerican	0.520***	0.589***
American	Andean America vs. Mesoamerican America	0.547***	0.554***
European	Andean Europe vs. Mesoamerican Europe	0.540***	0.654***
Andean	Andean America vs. Andean Europe	0.026***	0.033***
Mesoamerican	Mesoamerican America vs. Mesoamerican Europe	0.186***	0.276***

*** F_ST_ estimates significantly different from *P < 0.001* (1,000 permutations, Hudson 2000).

The AMOVA results showed that variation within gene pool within continent accounted for most (54%) of the genetic variance at the nuSSR loci (Table 4), and variance components between gene pools accounted for 46% of the total genetic variance (*P*<0.0001). Variance component between continents (America vs. Europe) was not significant.

**Table 4:** Hierarchical analysis of molecular variance (AMOVA) for 13 nuSSR markers in 345 Andean and Mesoamerican accessions of common bean from America and Europe.

**Source of variation**	**df**	**Sum of squares**	**Variance components**	**Percentage of totalvariance**	**SSR - ϕ**_PT_	***P*-value**
Between continents (America vs. Europe)	1	104.81	0.00	0	-0.333	n.s.
Between gene pools (Andean and Mesoamerican) within continent	2	1644.68	10.39	54	0.542	<0.0001
Among accessions within gene pool within continent	341	2994.18	8.78	46	0.389	<0.0001
Total	344	4743.66				

df: degree of freedom; significance tests with 10,000 permutations

### Analysis of the genetic structure and admixture detection

**Figure 2:**
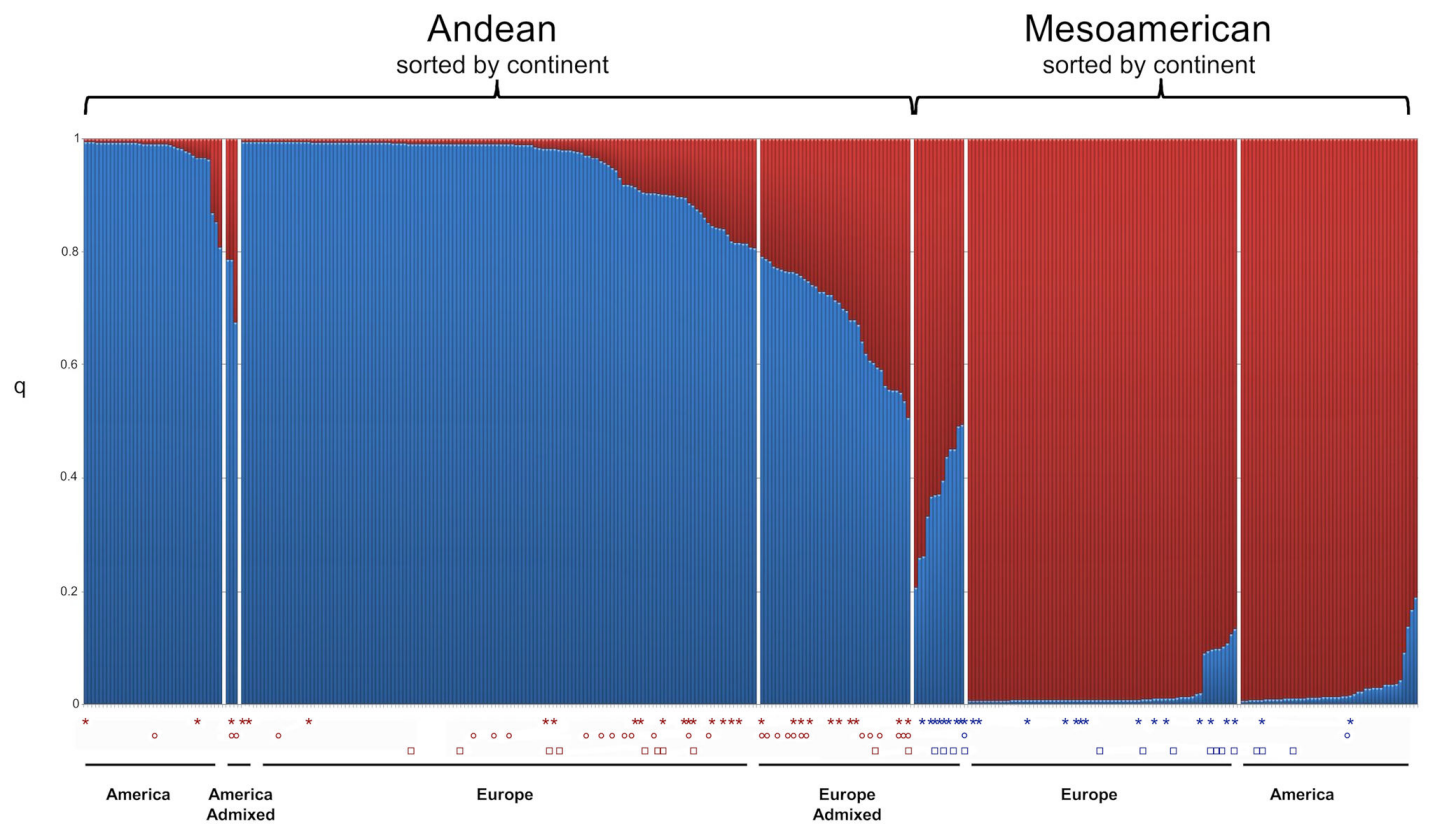
Population structure and membership fraction at K=2 for 345 Andean and Mesoamerican accessions of common bean from America and Europe estimated with STRUCTURE analysis for 13 nSSR and sorted by continent. Each accession is represented by a vertical histogram with two colors segments that represent the individual’s membership fraction in two clusters. The symbols under individual accessions indicate that the cpSSR haplotype (star) and/or *Pv-shatterproof1* allele (circle) and/or the Phaseolin type (square) are not in agreement with the Bayesian assignment to the gene pools (Andean vs. Mesoamerican) thus the accession is considered of hybrid origin.

Bayesian clustering of the information from the thirteen nuSSRs loci was used to identify distinct genetic populations, assign individuals to populations, and identify admixed individuals. The Evanno et al. [60] Delta K test suggested that our sample was made up of two main genetic groups (K = 2); cluster 1 included 216 accessions, while cluster 2 included 129 accessions. A bar graph of poster membership probabilities showed the two well-separated subgroups (Figure 2). A first inspection of the composition of the two clusters revealed a general correspondence with the gene pool of origin (Andean vs. Mesoamerican) based on cpSSR, phaseolin, and *Pv-shatterproof1* data. Indeed, cluster 1 included 188 accessions, with Andean cpSSR alleles, while cluster 2 included 105 accessions, with Mesoamerican cpSSR alleles (Table 5). For phaseolin marker, cluster 1 contained 203 accessions with Andean “T” and “C” phaseolin type (Table 5), while cluster 2 included 115 accessions with Mesoamerican “S” phaseolin type. For *Pv-shatterproof1* markers, cluster 1 contained 157 accessions with Andean alleles (Table 5), while cluster 2 included 120 accessions with Mesoamerican alleles (Table 5).

**Table 5:** CpSSR haplotype, phaseolin type and *Pv-shatterproof1* allele assignment to the two nuSSR clusters (Cluster 1 and 2) identified by STRUCTURE analysis in 345 American and European accessions of the common bean germplasm.

	**Type**	**All** (n = 345)		**America** (n = 89)		**Europe** (n = 256)	
**Marker**		Cluster 1	Cluster 2	Cluster 1	Cluster 2	Cluster 1	Cluster 2
		(Andean)	(Meso)	(Andean)	(Meso)	(Andean)	(Meso)
**CpSSR**	Andean	*188*	**24**	*40*	**1**	*148*	**23**
	Mesoamerican	**28**	*105*	**3**	*45*	**25**	*60*
**Phaseolin**	"T" type	*93*	**6**	*15*	**2**	*78*	**4**
	"C" type	*110*	**8**	*26*	**1**	*84*	**7**
	"S" type	**10**	*115*	*0*	*43*	**10**	*72*
	Not assigned	*3*	*0*	*2*	*0*	*1*	*0*
***Pv-shatterproof1***	Andean	*157*	**3**	*39*	**2**	*118*	**1**
	Mesoamerican	**28**	*120*	**3**	*44*	**25**	*76*
	Not assigned	*31*	*6*	*1*	*0*	*30*	*6*
**Total**		216	129	43	46	173	83
**Total hybrids**		**56**	**34**	**5**	**5**	**51**	**29**

Mismatch between at least one marker and Bayesian assignment identifies inter-gene pool hybrids.

n = sample size; marker type not assigned

Similarly to AMOVA and F_ST_, the Bayesian model-based cluster analysis at K = 2 failed to identify distinct differentiation between European and American accessions. Even when the K settings were increased beyond two population clusters, the accessions were divided into subpopulations based on gene pools of origin (data not shown).

### Identification of inter-gene pool hybrids

First, inter-gene pools hybrid genotypes were identified as those whose assignment based on cpSSR and/or phaseolin type and/or *Pv-shatterproof1* did not agree with the Andean vs. Mesoamerican Bayesian assignment based on 13 nuSSRs (i.e. genotype assigned by STRUCTURE to Andean gene pool but with Mesoamerica “S” phaseolin type).

As it is shown from the results of the STRUCTURE analysis (Figure 2) each accession is represented by a vertical histogram with two colors segments that represent the individual’s membership fraction in two clusters. The symbols under individual accessions indicate that the cpSSR haplotype (star) and/or the Phaseolin type (circle) and/or *Pv-shatterproof1* allele (square) are not in agreement with the Bayesian assignment to the gene pools (Andean vs. Mesoamerican) thus the accession is considered of hybrid origin.

In cluster 1 (Andean) 28 accessions (13.0%) showed Mesoamerican cpSSRs alleles, 10 accessions (4.6%) Mesoamerican “S” phaseolin type and 28 accessions (13.0%) Mesoamerican *Pv-shatterproof1* alleles (Figure 2). In cluster 2 (Mesoamerican) 24 accessions (11.1%) showed Andean cpSSRs alleles, 14 accessions (10.8%) Andean “T” or “C” phaseolin and 3 accessions (2.3%) Andean *Pv-shatterproof1* allele (Figure 2). Overall, in the European germplasm cpSSRs introgression (18.7%) was almost 4.1-fold higher than in the Americas (4.5%), phaseolin introgression (8.2%) was almost 2.5-fold higher than in the Americas (3.4%), and *Pv-shatterproof1* marker (10.2%) was almost 1.8-fold higher than that seen in the Americas (5.6%). Finally, using this approach, 90 out of 345 (26.1%) genotypes were identified that showed inter-gene pool admixture for at least one marker (cpSSR, phaseolin and/or *Pv-shatterproof1*) (Table 5).

Second, an interesting outcome from the model approach implemented in STRUCTURE software was also used: the admixture analysis interpreted based on the proportion of the genome of an individual originating from the different inferred clusters (genetic background matrix). We assumed that a genotype was only exclusively assigned to a particular genetic cluster if the population membership coefficient was higher than 0.80 (i.e. q ≥ 0.80); otherwise it was assumed to be jointly assigned to two clusters, probably due to admixture. Using this arbitrary threshold, 290 accessions out of 345 (84.1%) could be clearly divided into two distinct groups, while the other 55 accessions (15.9%) formed a mixed group (Figure 2). Admixture analyses of individual genotypes at the 0.8 cutoff identified 42 (19.4%) individuals that showed signals of hybridization (i.e. individuals with partial assignment to both clusters) among the Andean gene pool, and 13 (10.1%) among the Mesoamerican gene pool (Figure 2) (Table S1). Europe showed a higher proportion of putative hybrid individuals (n = 52, 20.3%) compared to the Americas (n = 3, 3.4%).

It was also observed that 31 out of 55 admixed genotypes had been already classified as hybrids because of mismatch of at least one of the three gene pool specific markers and the STRUCTURE assignment (Figure 2), while 24 new putative hybrid genotypes were detected only with the admixture analysis of nuSSRs. Thus, the total number of hybrids (n = 90) already identified should be increased by 24 putative hybrids that were identify with the Bayesian approach (Table S1).

Among the 256 European accessions, 153 accessions that were univocally attributed either to the Andean or to the Mesoamerican gene pool by all the three markers used and by the admixture analysis of simulated hybridization of STRUCTURE, were classified as “pure” (not hybrid); 103 accessions (69 Andean and 34 Mesoamerican) that showed mismatch between the chloroplast and the nuclear data were classified as putative hybrids. Thus, in Europe, the overall frequency of accessions derived from hybridization between the Andean and Mesoamerican gene pools was 40.2% (n = 103), 3.3 fold higher to that observed in the Americas (12.3%) (n = 11). Furthermore, in Europe, the proportion of hybrid individuals identified in the Andean (27.7%) was much higher than in the Mesoamerican (12.5%) group supporting the hypothesis of a preferred gene flow.

To further confirm inter gene pool hybridization, according to a previous study [16] we did calculate the frequency of recombinants in a sub-set of 220 European common beans, for which all the markers types (cpSSRs, phaseolin, *Pv-shatterproof1*) were available. 72 (32.7%) European genotypes that did not show for all three characters alleles from the same gene pool were considered hybrids. Supporting our marker-Bayesian combined hybrid detection approach, within the same sub-set of 220 European accessions previously studied, 72 accessions out of 74 (45 Andean and 29 Mesoamerican) were also identified as hybrids because they were Andean x Mesoamerican recombinants for three marker types (cpSSRs, phaseolin, *Pv-shatterproof1*) [16]. The remaining two did show correspondence for all three markers but were classified in a not matching group by STRUCTURE analysis and also classified as admixed.

In addition, combining hybrids identified by nuSSR admixture analysis of simulated hybridization of STRUCTURE (n= 43), we were able to identify more hybrid accessions that were observed as merely “pure” by the cpSSR analysis of Angioi et al. (2010). Indeed, 27 out of the 74 (45 Andean and 29 Mesoamerican) European accessions identified as putative hybrids, were also confirmed as hybrids by the admixed analysis of STRUCTURE using the earlier mentioned q threshold values, while 16 that were classified as “pure” (14 Andean, and 2 Mesoamerican) by marker recombination analysis were recognized as hybrids by nuSSR admixture analysis of simulated hybridization of STRUCTURE. Thus, in this subset of European germplasm the hybrid accessions were in total 90, more than previously detected.

Finally, as far as geographic distribution is concerned, inter-gene pool hybrid accessions were detected all over Europe, with higher frequencies in Central and Eastern Europe and lower frequencies in Spain and Italy (Table S1).

### Analysis of genetic diversity for “pure” accessions

To verify the effect of the presence of the hybrid accessions on the genetic structure of the European gene pools, 114 accessions out of 345 that were identified as inter-gene pool hybrid were discarded and the genetic diversity analyses were conducted using only the 231 “pure” accessions previously identified with the information provided by both chloroplast and nuclear markers and by STRUCTURE analysis. The summary statistics for the “pure” accessions were estimated (Table 2b) and compared with those obtained for the whole sample (Table 2a). When the putative hybrid accessions were excluded, and only the “pure” accessions were analyzed, the overall variation between the two continents for all calculated statistics remained substantially unchanged (no significant Wilcoxon signed-rank test, *P* >0.001 between whole sample and “pure” accessions). In contrast, differences between gene pools (Andean vs. Mesoamerican) within continent were more clear when only “pure” accessions were considered. Indeed, the relative deficit of allelic richness and gene diversity in Europe vs. America, calculated with the method defined by Vigouroux et al. [56], was always positive (Table 2b), with the Mesoamerican gene pool showing a five times larger loss of diversity (ΔH_e_ = 0.29) compared to the Andean gene pool (ΔH_e_ = 0.06). This shows that a genetic bottleneck did actually occur during common bean introduction in Europe and that it is made difficult to detect because of extensive inter-gene pool hybridization.

The F_ST_-based genetic differentiations between all the groups using only “pure” accessions are also shown in Table 3b. Excluding the putative hybrid accessions, the pairwise F_ST_ estimates showed, as expected, higher significant genetic differentiation between all the groups, supporting the genetic diversity data.

## Discussion

In this study diversity of the common bean within and between Mesoamerican and Andean gene pools was compared in landraces from America and from Europe, to elucidate the effects of bottleneck caused by its introduction into Europe, and of selection for adaptation during the subsequent spread of the common bean over the whole of Europe. The information obtained using nuSSR markers, along with phaseolin, cpSSR and *Pv-shatterproof1* locus polymorphism data available from previous studies [16,27], allowed the following conclusion.

First, the nuSSR results confirmed the genetic structure of the European common bean germplasm, and specifically that both the Andean and the Mesoamerican gene pools are present in Europe, as described earlier by various authors using different markers [16,27]. The separation of European common bean landraces into the two recognized gene pools was confirmed in this study by PCoA, where the Andean and Mesoamerican gene pools are clearly distinct along the first axis (PC1), and by STRUCTURE analysis, which revealed that 216 landraces (62.6%) were present in group 1, and 129 (37.4%) in group 2, recognized respectively as the Andean group and the Mesoamerican group.

Second, the overall genetic diversity (H_e_) was 0.529, indicating that the common bean landraces used in this study displayed a substantial genetic diversity. Since estimates of genetic diversity are not affected by differences in sample size, direct comparison between different studies that also include different accessions are possible. Our findings were in accordance with the results of an earlier study on nuSSRs diversity in the common bean (H_e_=0.527) [61], but larger than estimates based on cpSSRs (H_e_=0.45) [16]. These results can be explained by considering the contrasted patterns of inheritance and the different ploidy level of nuclear and chloroplast markers [62].

Third, the amount of genetic diversity was not different between America (H_e_ = 0.52) and Europe (H_e_ = 0.51), as was also observed for cpSSR markers [16] (America H_e_ = 0.46 and Europe H_e_ = 0.45), apparently in contrast with the hypothesis of significant bottleneck [18]. But, in disagreement what was observed for cpSSRs, where diversity within gene pool in America (Andean H_e_ = 0.32 and Mesoamerican H_e_ = 0.34) was almost the same as in Europe (Andean H_e_ = 0.36 and Mesoamerican H_e_ = 0.34) [16], nuSSRs showed similar values in America (Andean H_e_ = 0.33 and Mesoamerican H_e_ = 0.31), but much larger values in Europe (Andean H_e_ = 0.36 and Mesoamerican H_e_ = 0.29). Thus, the analysis of the overall genetic diversity not only supported the hypothesis of an overall not significant bottleneck both for nuSSRs and cpSSRs but surprisingly, when the two gene pools were analyzed separately, nuSSRs actually showed a significant increase of diversity in Europe.

Fourth, the genetic differentiation between continents was low for both nuSSRs (F_ST_ = 0.049; *P* < 0.001) and cpSSRs (F_ST_ = 0.024; *P* < 0.001), with nuSSRs F_ST_ two times larger than cpSSR F_ST_. One possible interpretation for this observation has been proposed by Petit et al. [63]: cpSSR is effectively haploid, and its effective population size is smaller than the one for nuclear gene. Hence, F_ST_ values at cpSSR markers will reach equilibrium faster than F_ST_ values at nuclear genes, resulting in transient situations where F_ST_ of maternally inherited markers is lower than F_ST_ of nuclear gene.

Fifth, to estimate the amount of hybridization that occurred in Europe between the two common bean gene pools, the analysis of recombinant frequency based on cpSSRs, phaseolin, and *Pv-shatterproof1* vs. nuSSRs STRUCTURE assignment, and the admixture analysis of simulated hybridization, were combined to distinguish between “pure” and hybrid genotypes. Our results clearly demonstrate the presence of a larger percentage of intermediate genotypes (hybrids) in Europe (40.2%) than in the Americas (12.3%), emphasizing the power of this approach.

Using merely the Bayesian assignments based on nuSSRs, we could confirm only 25 (34.7%) of the European hybrid genotypes previously identified by [16], whereas combining the previously used marker systems (cpSSR, phaseolin, and *Pv-shatterproof1*) with the nuSSR data, we were able to identify a number of intermediate genotypes very close to what was previously only estimated by [16]. Combining the nuclear SSRs, which are more polymorphic and bi-parentally inherited, with the haploid cpSSRs, which are non-recombinant and usually maternally inherited, permits access to different parts of the history of populations: nuclear SSRs are suitable for studying recent and local evolutionary processes, while cpSSRs are effective indicators of population subdivision and differentiation for tracing ancient divergences [64]. The use of only nuSSR data is more likely to underestimate genetic admixture, because introgressed markers may be counted as part of the normal gene pool of either group. Thus, the estimation of the actual rate of hybridization proved to depend highly on the markers used. For instance, in this study, when all the available information was used, 103 inter-gene pool hybrid genotypes (40.2%) were detected in the European core collection, while the number of hybrids detected using each marker or analysis separately was: a) 21 using only phaseolin (8.2%); b) 26 based on *Pv-shatterproof1* analysis (10.1%); c) 48 based only on cpSSRs (18.7%); and d) 52 based on STRUCTURE analysis (20.3%). The estimated number of hybrids thus ranges from 8.2% to 40.2%, which is a five-fold difference between estimates, depending on markers and/or method used. Thus, the disagreements among markers or method observed in our data confirm the necessity of multi-marker approaches to the study of hybridization.

Sixth, compared to America, where the frequency of inter-gene pool hybrids is 12.3%, almost all of which reside within the Mesoamerican germplasm, in the European common bean germplasm, 40.2% of the accessions show evidence of introgression, and the frequency is higher in the Andean gene pool (27.7%) than in the Mesoamerican gene pool (about 12.5%) supporting the hypothesis of a preferred gene flow.

Hybridization among gene pools has also been described in common bean germplasm from various secondary centers of diversity. In Brazil, comparing nuSSRs to phaseolins, Burle et al. [65] found very limited introgression (4%) in spite of the widespread sympatry between the two gene pools. In Ethiopia and Rwanda [66,67], only 1 and 10% of hybrids, respectively, were found (considered as individuals intermediate among gene pools in the neighbor-joining tree), while, in a collection of Chinese landraces, Zhang et al. [68] found 5% hybrids, and noted that average seed weight of the Andean types was lower than that of the American Andean beans, with the opposite for the Mesoamerican Chinese bean.

Seventh, hybridization between the Andean and Mesoamerican gene pools in Europe has had a significant impact on the maintenance of the overall amount of genotypic diversity. This view was largely supported in the present study by findings that “pure” European common bean landraces harbored lower genetic diversity (in terms of total number of alleles, allelic richness, Shannon’s information index, and gene diversity) than American common bean. Thus introgression appears to be responsible for much of the genetic diversity observed in the European germplasm. When only the “pure” accessions were considered, the reduction of genetic variation following the introduction of common bean into Europe was statistically significant as a whole, and also for the Andean and Mesoamerican gene pools when analyzed separately.

Indeed, our results indicate that, when the two “pure” gene pools were considered as a whole, the genetic bottleneck that followed the introduction of common bean into Europe was 16% (ΔR_s_) in terms of relative loss of alleles and 2% (ΔH_e_) for reduction of genetic diversity, whereas, when the two “pure” gene pools were considered separately, the deficit in the European germplasm was 5% (ΔR_s_) and 6% (ΔH_e_) for the Andean gene pool, and 10% (ΔR_s_) and 29% (ΔH_e_) for the Mesoamerican. Therefore, loss of diversity in Europe was higher for the Mesoamerican gene pool than for the Andean gene pool. This suggests that not all the variation available in America for Mesoamerican common bean was introduced into Europe or, if introduced, was selected against during the adaptation to the new environmental conditions. This result is in agreement with what was suggested by Logozzo et al. [27] for a European “core collection”, where the mean seed size and weight was larger in Europe than in America for the Mesoamerican germplasm.

The implication of the foregoing is that introgression among gene pools did influence the evolution of common bean of the European gene pool. This is very important since breeders and geneticists always search for genotype with unique genes or gene combinations. Unfortunately, despite the fact that inter-gene pool crosses could provide increased level of genetic variation, when American germplasm was used, incompatibility caused by lethal genes was observed [2] and recombinants have proven not successful, phenotypic abnormalities and poor performance in the progeny were observed [7] perhaps due to the disruption of co-adapted sets of genes. The high frequency of European inter-gene pool hybrids demonstrates that introduction in Europe was an exceptional evolutive opportunity for common bean, where a large number of landraces were differentiated to adapt to a highly variable environment and to fulfill the highly diversified needs of European farmers.

Along with possible proximity within the farm field that favored the high frequency of inter-gene pool crossing, the partial reproductive isolation that was present in the American germplasm might have been mitigated by the significantly large introduction bottleneck for Mesoamerican germplasm. This possibly suggests that some American races were less represented in the germplasm introduced in Europe. The European germplasm is therefore more complex than previously thought, and contains additional molecular diversity that remains to be explored for genetic and breeding proposes.

## Conclusions

The genetic data presented here confirmed the occurrence of extensive hybridization between the Andean and Mesoamerican gene pools in Europe. Combining two different approaches we have shown at the molecular level, that 103 common bean genotypes from different European germplasm accessions contain detectable admixture. The frequent inter-gene pool hybridization in the European common bean, as a result of adaptation to the new environmental conditions, might have mitigated the effects of the bottleneck of introduction in Europe that was significant and affected the Mesoamerican gene pool five times more than the Andean. Furthermore, introgressions between the gene pools might have created new interesting combinations of traits, such as higher adaptability to environmental stresses, diseases or insects [69], and might have helped to break the negative associations between seed weight and yield potential [70]. For these reasons, the European germplasm of the common bean appears to be of great importance for breeding, which often aims to recombine Andean and Mesoamerican traits, as it may circumvent some of the difficulties in transferring traits between the two gene pools.

## Supporting Information

Table S1 **Accessions used in this study and Structure membership coefficient for K=2.**
(XLSX)
